# The Effect of Exercise on Inflammatory Markers in PCOS Women: A Systematic Review and Meta-Analysis of Randomized Trials

**DOI:** 10.1155/2023/3924018

**Published:** 2023-02-09

**Authors:** Mozhgan Hafizi Moori, Saeed Nosratabadi, Naghmeh Yazdi, Razieh Kasraei, Zeinab Abbasi Senjedary, Razieh Hatami

**Affiliations:** ^1^Department of Midwifery, Faculty of Nursing and Midwifery, Ahvaz Branch, Islamic Azad University, Ahvaz, Iran; ^2^Department of Nutrition, Electronic Health and Statistics Surveillance Research Center, Science and Research Branch, Islamic Azad University, Tehran, Iran; ^3^Department of Persian Medicine and Pharmacy, Ahvaz Jundishapur University of Medical Sciences, Ahvaz, Iran; ^4^Department of Traditional Medicine, School of Persian Medicine, Tehran University of Medical Sciences, Tehran, Iran; ^5^Department of Midwifery, Nursing and Midwifery School, Tehran Medical Sciences Islamic Azad University, Tehran, Iran

## Abstract

**Background:**

Polycystic ovary syndrome (PCOS) is a typical female disorder that influences different aspects of women's health. There is a direct association between inflammation and PCOS status. Some evidence supports the beneficial effects of exercise on inflammation status in PCOS women, while others cannot find a significant association. This study aimed to clarify the effect of exercise on inflammatory markers in women with PCOS.

**Method:**

Electronic searches in international databases were performed to identify eligible publications up to August 2021, which compared the effects of exercise on inflammatory markers in the intervention group compared to the control group in PCOS women. A weighted mean difference (WMD) using a random-effect model was applied for reporting results.

**Results:**

A total of 2525 records were found via database searching, of which 10 were eligible to be included in the analysis. The results of the meta-analysis revealed that exercise could significantly lower the serum level of CRP (WMD: −0.43 mg/L; 95% CI: −0.66 to −0.21; *P* ≤ 0.01; *I*^2^: 84.9%; *P* < ≤0.01), while it was not considerable for adiponectin (WMD: −0.33 *μ*g/mL; 95% CI: −0.97 to 0.31; *P*=0.30; *I*^2^: 0%; *P*=0.97). In addition, subgroup analyses indicated a significant effect of exercise on CRP in individuals ≥30 years, sample size ≥15 individuals, and aerobic training.

**Conclusion:**

Exercise training can reduce CRP levels in women with CRP, particularly in women older than 30 years of age, and in studies with more than 15 participants. The effect of exercise on adiponectin was not noticeable.

## 1. Introduction

Polycystic ovary syndrome (PCOS) is a female disease involving women of reproductive age [1]. It is the most prevalent endocrine disorder, so about 4–21% of women deal with it [2]. This hormonal anomaly results from a disturbance in sex hormones, leading to irregularity in the menstrual cycle and anovulation and eventually infertility [3]. Hirsutism, acne, male pattern alopecia, and hyperandrogenism are other clinical manifestations of PCOS [4]. In addition, women with PCOS are at higher risk of obesity, type 2 diabetes, cardiovascular diseases, and ovarian and endometrial cancers [5].

A wide range of genetic and environmental factors contributed to the development of PCOS [6]. It is characterized by two main symptoms: abnormal fat accumulation and insulin resistance [7]. Visceral adipose tissue releases inflammatory cytokines that influence ovarian follicular function [8]. Moreover, there is a direct association between low-grade inflammation, high level of androgens, and PCOS, which result in higher fat accumulation [9]. High levels of inflammatory biomarkers, such as tumor necrosis factor-*α* (TNF-*α*) and interleukine-6 (IL-6), are associated with insulin resistance, a frequent disturbance in this syndrome [10]. Besides, other researchers also found that, due to obesity in PCOS patients, the increased circulating leptin (LP) levels within the body led to leptin resistance, which can also significantly promote chronic low-grade inflammation [11].

So far, there is no complete cure for PCOS, but it can be effectively managed [12]. Some guidelines recommend lifestyle modification alongside pharmaceutical intervention for the long-term control of PCOS in involved patients [12]. Due to concerns regarding the side effects of medications, many women with PCOS −about 70%−seek out different complementary medicine modalities [13]. Diet, physical activity, and using herbal medicine are popular nonpharmacological interventions in PCOS management [14, 15]. For example, the extract of some herbs such as Galega officinalis, celery, and cinnamon, due to their high antioxidant concentration, can neutralize oxidative stress, which improves ovarian function [16, 17]. Nutritional strategies such as moderate weight loss, high monounsaturated and low saturated fatty acids diet rich in fiber, and low glycemic index diet can have protective effects on PCOS [18, 19].

Previous studies have approved the beneficial effects of exercise on insulin resistance [20]. Its anti-inflammatory properties have also been demonstrated in different conditions [21]. However, they depend on the type of exercise, illness, consumed medication, and baseline inflammation [22]. It seems that appropriate exercise can be helpful in the prevention and treatment of several metabolic outcomes in PCOS [23]. Several meta-analyses depicted that exercise had a beneficial impact on inflammation in people with chronic diseases, such as breast cancer [24], heart disease [25], and diabetes [26], and there is no concord about the effect of exercise on the circulating inflammatory biomarkers in the women with PCOS. In an intervention by Almenning et al., the findings did not reveal that exercise had a positive effect on the inflammatory biomarkers in PCOS patients (reference), while others failed to present such results [27].

Due to these discrepancies, the present study was carried out to summarize the evidence of the effects of exercise on the levels of CRP, TNF-*α*, IL-6, adiponectin, and leptin in women with PCOS via a systematic review and meta-analysis of randomized controlled trials.

## 2. Method

This study was conducted following the guidelines of the PRISMA (preferred reporting items for systematic reviews and meta-analysis) statement.

### 2.1. Search Strategy

We performed a literature search using Medline's online databases (PubMed) and Scopus for related publications up to August 2021. To find relevant publications, use the following medical subject headings (MeSH) and nonMeSH keywords, such as (leptin or adipokines or interleukin-6 or tumor necrosis factor-alpha or interleukin-8 or interleukins or C-reactive protein) AND (polycystic ovary syndrome or PCOS) AND (exercise or exercise tolerance or exercise therapy or resistance training or muscle stretching exercises or high-intensity interval training or endurance training). The search strategy in the PubMed database was represented in a Supplementary Table (available (here)). We did a systematic search of the above database for systematic reviews and manually checked the list of references to identify studies that may have been lost. In addition, duplicate studies as well as unpublished studies were excluded.

### 2.2. Selection of Studies

Following the elimination of duplicate articles, a researcher evaluated the results of the research. Subsequently, the selected studies were retrieved and studied by two researchers. Any disagreement between the two researchers was resolved by discussion or by a third party.

### 2.3. Inclusion and Exclusion Criteria for Studies

In our meta-analysis, eligible issues were included based on these criteria: (1) studies that used a clinical trial design; (2) those studies that investigate exercise, (3) studies that report inflammation factors associated; (4) human studies; and (5) women with PCOS. Studies that met the following criteria were excluded: (1) studies on children and animals; (2) nonoriginal research (letters, review articles, and meta-analysis); (3) insufficient data is available; and (4) studies without exercise or control group.

### 2.4. Data Extraction

The following data of interest from each study were extracted: first author, year of publication, study population, sample size, age, weight, and BMI (Table 1), exercise details (type of exercise, intensity, exercise frequency, session duration, and intervention duration), and the measure of inflammation factors (interleukin-6, tumor necrosis factor-alpha, interleukin-8, and C-reactive protein), leptin, and adiponectin. For some studies that graphically presented data, mean and standard deviation were extracted using the GetData Graph Digitizer 2.24.

### 2.5. Assessment of Study Quality

Study quality and systematic bias assessment in the included studies were assessed by Cochrane criteria (25). These items were defined for each study: (1) random sequence generation, (2) allocation concealment, (3) blinding of participants and personnel, (4) blinding of outcome assessment, (5) incomplete outcome data, (6) selective outcome reporting, and (7) other potential sources of bias. According to the recommendations of the Cochrane guidebook, we have three judgments for each item: low risk of bias, high risk of bias, and unclear risk of bias, which indicate insufficient information. If two or more criteria are listed as having a high or unclear risk of bias, the study has poor quality. Hence, we designed the quality of publications according to the Cochrane criteria and reported it in Table 2.

### 2.6. Data Synthesis and Statistical Analysis

This analysis was conducted using Stata software version 20 (Stata Corp., L.P, College Station, TX, USA). Random and fixed effects models were applied to obtain pooled estimates of exercise impacts on liver functions using the weighted mean difference (WMD). Studies that reported two or more nutritional interventions were entered as separate studies. Finally, nine studies were included to compare the effect of exercise with the control group on inflammation factors in women with PCOS. The mean change in items under the survey was calculated based on differences between the baseline and final data. Some studies established a standard error of the mean, from which we calculated the standard deviation according to the formula (SD = SEM × square root of *N*), where *N* means sample size. Then, we calculated the SD of the mean difference as follows: SD change = square root of ((SDbaseline^ 2 + SDfinal ^ 2) − (2 × *R* × SD baseline × SD final)). SD of mean differences was calculated using a correlation coefficient “*R*” of 0.9. When the publications revealed medians and ranges or 95% CIs, the mean was calculated. The between-trial WMD and 95% CI were calculated. Between-study heterogeneity was examined using the *I*-square (*I*^2^) test. Publication bias was assigned by visual assessment of the funnel plot and Egger's test. *P* values <0.05 were considered notable.

## 3. Results

### 3.1. Included Studies

**Figure 1:**
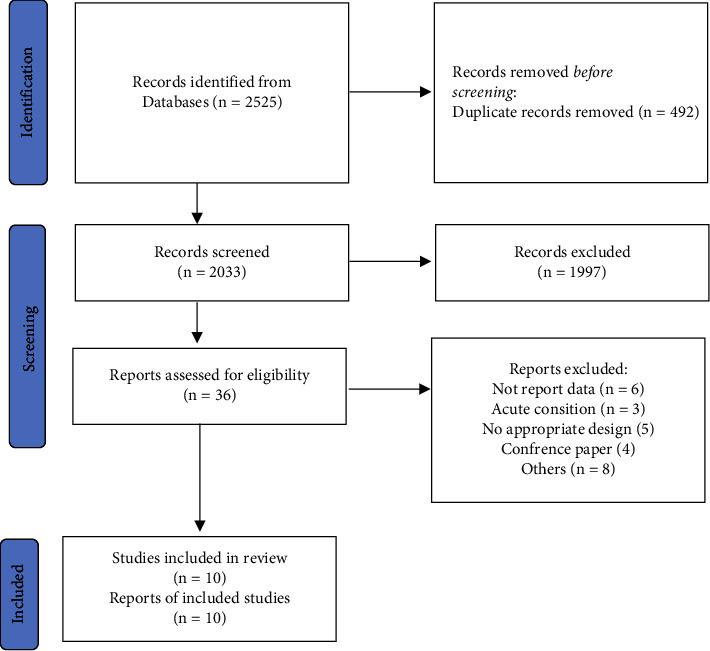
Summary of the search strategy and selection process based on included and excluded studies.

A total of 2525 articles were identified through the PubMed and Scopus databases, and after the removal of duplicates, a total of 2033 articles remained. In addition, we found an additional essay in the reference lists of the manuscripts retrieved. After the articles were eliminated based on the eligibility criteria, 10 articles remained (Figure 1). In all studies, except one that included a diet, the control group did not receive any supplements or diets.

### 3.2. Study Characteristics and Quality Assessment

A total of 540 participants (with a mean age of 27.3 years) had been enrolled in the studies, all of whom were women. Age was not reported in the article. Of the fifteen studies (10 papers) in the meta-analysis, four articles were exclusively conducted on overweight women, three papers were conducted solely on obese women, one was conducted on normal-weight women, and one was not reported. The mean BMI of the participants was 30.2 kg/m^2^.

### 3.3. Outcomes of a Systematic Review

Among the included studies, CRP was reported in 10 studies, adiponectin in 6 studies, IL-6 and TNF-a in 2 studies, and leptin in 1 study. However, only CRP and adiponectin entered the meta-analysis. Since the studies entered did not have enough information for IL-6, TNF-a, and leptin, they were not included in the meta-analysis and were presented only as a systematic review.

One study showed that TNF-a in the intervention group decreased compared to the control group, but no change in IL-6 was observed [28]. However, in another study, a negative association between exercise and IL-6 and TNF-a was observed [22]. Almenning et al.'s investigation found no association between exercise and leptin [27].

### 3.4. Outcomes of Meta-Analysis

**Figure 2:**
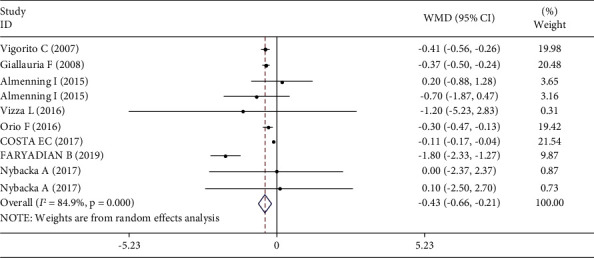
Forest plot for CRP studies (*n* = 10). The graph depicts WMD and 95% CI for individual studies and the pooled estimate.

**Figure 3:**
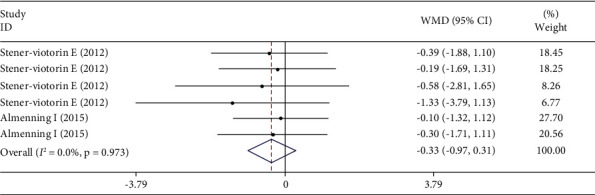
Forest plot for adiponectin studies (*n* = 6). The graph depicts WMD and 95% CI for individual studies and the pooled estimate.

The pooled effect size of 10 studies (8 articles) demonstrated a significant decrease in CRP (*p* ≤ 0.01) following the intervention (Figure 2). In contrast, all changes in adiponectin (*p*=0.30) were not statistically significant (Figure 3). In addition, between-study heterogeneity was significant for CRP, but there was no significance for adiponectin.

### 3.5. Subgroup Analysis for CRP

Subgroup analysis was performed for age (<30 years vs. ≥30 years), BMI (normal and overweight vs. obese), duration (<3 months vs. ≥3 months), sample sizes (<15 individuals for each group vs. ≥15 individuals for each group), and type of exercise (aerobic vs. resistance vs. combined). Subgroup analyses indicated a significant effect of exercise on CRP in individuals ≥30 years (*p* ≤ 0.01), sample size ≥15 individuals (*p* ≤ 0.01), and aerobic training (*p* ≤ 0.01) but did not have a significant effect on individuals <30 years, sample size <15 individuals, resistance training, and combined exercises. However, BMI and duration did not explain the heterogeneity seen between studies for analyses of CRP.

### 3.6. Sensitivity Analysis and the Risk of Bias

**Figure 4:**
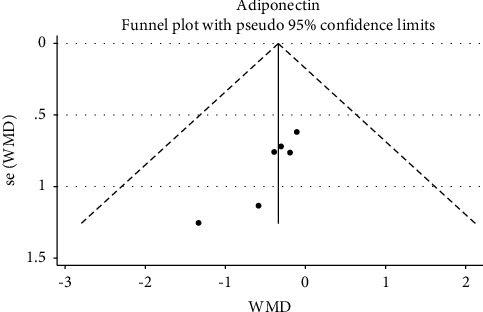
Funnel plot for evaluating publication bias in adiponectin.

**Figure 5:**
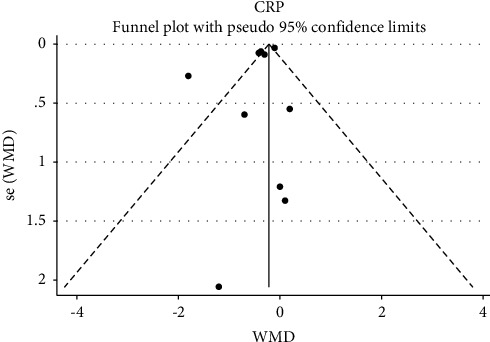
Funnel plot for evaluating publication bias in CRP.

Visual assessment of the funnel plots indicated no publication bias for CRP and adiponectin (Figures 4 and 5). Egger's test also did not provide evidence of publication bias for CRP (*P*=0.18), but there was evidence of publication bias for adiponectin (*p*=0.01).

Sensitivity analysis (influence analysis) performed for CRP and adiponectin demonstrated that any studies were not outliers.

## 4. Discussion

This systematic review and meta-analysis study investigated the effects of exercise on inflammatory markers in PCOS. Overall, our results demonstrated that training is effective in lowering serum CRP levels compared to the control group. Also, subgroup analysis showed that exercise decreased CRP in individuals ≥30 years, sample size ≥15 individuals, and aerobic training significantly compared to the control group. However, this study did not observe any significant changes in the serum level of adiponectin in women with PCOS.

Pooled results of the current study demonstrated the positive effects of exercise on inflammatory biomarkers. In another meta-analysis by Rose et al. [29] that the effect of exercise intensity on chronic inflammation was investigated, findings failed to establish the effectiveness of exercise on inflammation in the overall analysis, but subgroups indicated positive impacts of intensity exercise on CRP, middle-aged adults, and intervention with higher than 9 weeks. However, in one study done by Costa et al. TNF-a in the exercise group decreased compared to the control group, but no change in IL-6 was observed [28]. In addition, a study conducted by Dantas et al. demonstrated that exercise leads to a decrease in IL-6 and TNF-a [22].

Furthermore, this study did not observe significant efficacy in the effects of exercise on adiponectin. It seems that there is an inverse association between adiponectin concentration and sympathetic activity in women with PCOS, which could be considered as an indicator of PCOS alongside insulin resistance [30]. In line with the present results, Almenning et al.'s study indicated that neither strength training nor high-intensity interval training affected the serum levels of adiponectin and leptin [27]. While basic military training for 8 weeks could improve adiponectin concentration in healthy young men.

Within this meta-analysis, there was significant heterogeneity in the type of exercise and age of individuals. Based on the present findings, aerobic training is more effective in the reduction of inflammation in PCOS women, especially in individuals over 30 years. In a study conducted by Boeno et al. and colleagues [31] in order to compare the effects of aerobic and resistance exercise on middle-aged hypertensive patients, results revealed that although both types of training can be effective in declining blood pressure and improving endothelial function, only aerobic training decreased inflammatory markers like CRP, vascular cell adhesion molecule-1, and lectin-like oxidized LDL receptor-1. On the other hand, a study stated that resistance exercise could play a positive role in lowering inflammatory markers such as NF-*κ*B, IFN-*γ*, and eotaxin-1 after 12 weeks of intervention; however, it should be taken into account that the population was elderly obese women [32].

In addition, an article demonstrated that aerobic exercise, but not resistance exercise, reduces inflammation (serum IL-18, CRP, and IL-6) [33]. Also, a study conducted by Silverman et al. indicated that aerobic exercise reduces inflammation during weight loss in overweight postmenopausal women [34]. In contrast to our study, one study observed that the increase in inflammation after adjuvant radiation therapy in breast cancer patients was counteracted by progressive resistance exercise [35]. In another study, it was observed that aerobic physical exercise compared to the control group had no significant effect on the level of inflammation, especially CRP [36]. Another study conducted in 2017 found that combined exercise training (aerobic and resistance) attenuates inflammation in obese postmenopausal breast cancer survivors [37]. This difference in results is most likely due to differences in the study population.

Moreover, in healthy young men, Ihalainen et al.'s [38] study showed that exercise significantly reduced circulating TNF-*α*, hs-CRP, leptin, resistin, and MCP-1. Also, it was revealed that there are related changes in abdominal fat mass and this inflammation marker. This study reported that the decreases in inflammation factors might be correlated with reductions in abdominal fat mass [38]. In this regard, our study showed that exercise, compared to the control group, reduced CRP levels while not showing a significant change in adiponectin. It seems that this change in inflammation in women with PCOS is not due to a reduction in abdominal fat. If it was related to the loss of abdominal fat, the adiponectin also had to be changed. There may be more reasons that could play a role. Similar to the current study, the Shavandiet al. study showed that exercise has beneficial effects on inflammation status without any influences on body weight or serum lipid levels [39]. Also, another study by Ryan et al. showed that exercise plus weight loss, compared with weight loss alone, reduced inflammation in obese postmenopausal women. This article is like our study, which recommends that physical activity is a necessary component of lifestyle modification, especially in women [40].

Some of the possible mechanisms that explain the effect of exercise on inflammation include (1) stimulating the accumulation of anti-inflammatory cytokines (IL-10 and IL-1 receptor antagonist), (2) alterations of psychosocial factors (depression, stress, and anxiety) (3) adrenergic receptors expressed on several tissues (leukocytes, adipose tissue, and muscle), and (4) weight loss and reducing visceral fat [33, 38].

The strength of this study is that it is the first to evaluate the effects of exercise on inflammatory markers in women with PCOS. Nevertheless, one of our study's limitations was that some inflammatory markers (IL-6, TNF-a, and leptin) did not have enough paper for a meta-analysis, so it was reported as a systematic review. In addition, considering that one of the questions used to assess the quality of studies in the Cochrane Risk of Bias questionnaire is the blinding of participants, this question cannot be answered for our research because people who exercise cannot be blinded. Moreover, as this study was conducted outside of England, we were not able to register it in PROSPERO because it took a long time.

In conclusion, this meta-analysis indicated that exercise could have beneficial effects on levels of CRP. Regarding the restricted number of investigations, more interventions with a larger sample size are required to deepen our understanding of exercise effects on additional inflammatory markers in women with PCOS.

## Figures and Tables

**Table 1:** Characteristics of studies included in this systematic review.

First author	Year	Country	Type of exercise	Mean age (year)	Mean BMI	Duration	Sample size I	Sample size C
Vigorito	2007	Italy	Aerobic	I: 21 C: 21	I: 29.2 C: 29.5	3 month	45	45
Giallauria	2008	Italy	Aerobic	I: 22 C: 22	I: 27.4 C: 26.5	3 month	62	62
Almenning	2015	Norway	Resistance	27	I: 26.1 C: 26.5	10 weeks	8	9
Almenning	2015	Norway	Aerobic	27	I: 41.3 C: 33.8	10 weeks	8	9
Vizza	2016	Australia	Resistance	I: 26 C: 29	I: 26.7 C: 27	12 weeks	7	6
Orio	2016	Italy	Aerobic	I: 25 C: 26	I: 32 C: 33.6	6 month	39	50
Costa	2017	Brazil	Aerobic	I: 27 C: 24	I: 21.1 C: 21.3	16 weeks	14	13
Faryadian	2019	Iran	Combined	I: 34 C: 32	I: 38.1 C: 35.4	12 weeks	12	12
Nybacka	2017	Sweden	Combined	I: 31 C: 29	I: 34.8 C: 35.4	4 month	12	14
Nybacka	2017	Sweden	Combined	I: 31 C: 29	I: 29.2 C: 29.5	4 month	17	19
Stener-victorin	2012	Sweden	Aerobic	NR	NR	32 weeks	30	15

In: intervention; C: control; NR: not reported.

**Table 2:** Risk of bias in the assessment of the studies.

Study	Random sequence generation	Allocation concealment	Blinding of participants and personnel	Blinding of outcome assessment	Incomplete outcome data	Selective outcome reporting	Other sources of bias
Vigorito	L	L	—	L	L	U	L
Giallauria	U	U	—	L	L	L	L
Stener-victorin	L	L	—	L	H	L	L
Almenning	L	L	—	L	H	L	L
Vizza	L	L	—	U	H	L	L
Orio	L	L	—	L	L	U	L
Costa	L	L	—	U	L	L	L
Faryadian	H	H	—	U	L	L	U
Nybacka	L	L	—	U	H	L	U

U: unclear risk of bias; L: low risk of bias; H: high risk of bias.

## Data Availability

The data used to support the finding of this study are available from the corresponding upon request.

## Supplementary Materials

Supplementary Materials Supplemental Table: Terms used to search articles on the effect of exercise on inflammatory markers in PCOS women.
