# Supplementary material for: The Effect of Exercise on Inflammatory Markers in PCOS Women: A Systematic Review and Meta-Analysis of Randomized Trials
Source: Int J Clin Pract. 2023 Feb 9;2023:3924018. doi: 10.1155/2023/3924018 (PMC9934983; doi:10.1155/2023/3924018)
Supplement: Supplementary Materials — Supplemental Table: Terms used to search articles on the effect of exercise on inflammatory markers in PCOS women. [file 3924018.f1.docx]

**Supplemental Table:** Terms used to search articles on the effect of exercise on inflammatory markers in PCOS women

| **Concept 1** | ("Leptin"[Mesh] OR "Adipokines"[Mesh] OR "Interleukin-6"[Mesh] OR "Tumor Necrosis Factor-alpha"[Mesh] OR "Interleukin-8"[Mesh] OR "Interleukins"[Mesh] OR "C-Reactive Protein"[Mesh] OR "Interleukins"[TIAB] OR "Tumor Necrosis Factor-alpha"[TIAB] OR "C-Reactive Protein"[TIAB]) |
| --- | --- |
| **Concept 2** | ("Exercise"[Mesh] OR “Exercise"[TIAB] OR "Exercise Tolerance"[Mesh] OR "Exercise Tolerance"[TIAB] OR "Exercise Therapy"[Mesh] OR "Exercise Therapy"[TIAB] OR "Exercise Test"[Mesh] OR "Exercise Test"[TIAB] OR "Resistance Training"[Mesh] OR "Resistance Training"[TIAB] OR "Muscle Stretching Exercises"[Mesh] OR "Muscle Stretching Exercises"[TIAB] OR "Breathing Exercises"[Mesh] OR "Breathing Exercises"[TIAB] OR "High-Intensity Interval Training"[Mesh] OR "High-Intensity Interval Training"[TIAB] OR "Endurance Training"[Mesh] OR "Endurance Training"[TIAB]) AND ("Clinical Trials as Topic"[Mesh] OR "Clinical Trial" [Publication Type] OR "Clinical Trials as Topic"[tiab] OR "Clinical Trial" [tiab] OR "intervention"[tiab] OR "controlled trial"[tiab] OR "randomized"[tiab] OR "randomised"[tiab] OR "random"[tiab] OR "randomly"[tiab] OR "placebo"[tiab] OR "assignment"[tiab])) |
| **Concept 3** | (pcos[tiab] OR ( (polycystic[TIAB] OR cystic[TIAB] ) AND ovar*[TIAB]) OR "Polycystic Ovary Syndrome"[Mesh] OR (Sclerocystic [TIAB] AND Ovar*[TIAB])) |
| **Search** | Concept 1 AND Concept 2 AND Concept 3 |
